# Utilisation of Home Laundry Effluent (HLE) as a catalyst for expeditious one-pot aqueous phase synthesis of highly functionalised 4-thiazolidinones

**DOI:** 10.1186/2193-1801-2-466

**Published:** 2013-09-16

**Authors:** Udaya Pratap Singh, Hans Raj Bhat, Mukesh Kumar Kumawat, Ramendra K Singh

**Affiliations:** Department of Pharmaceutical Sciences, Sam Higginbottom Institute of Agriculture Technology and Sciences, Formerly Allahabad Agricultural Institute, Deemed to be University, Allahabad, 211007 India; Anand College of Pharmacy, Agra, 282007 India; Nucleic Acids and Antiviral Research Laboratory, Department of Chemistry, University of Allahabad, Allahabad, 211002 India

**Keywords:** Home laundry effluent, One-pot synthesis, 4-thiazolidinones

## Abstract

**Background:**

The impact of global warming and associated climate changes have built up pressure to focus on the option of green chemistry over traditional one for long term sustainability of the environment. Considering the fact, for the first time, efficient HLE catalysed expeditious one-pot synthesis of highly functionalised 4-thiazolidinones has been developed.

**Results:**

These hybrid molecules were synthesized in good to excellent yields. The ease of work-up of the reactions less time required and mild conditions are notable features of this protocol. It was inferred that halogen containing derivatives were well suited to this condensation-cyclization reaction with varying rates to afford 4-thiazolidine derivatives. In general, the substitution on the aldehyde part was shown as a main determinant for reaction time and the product yield.

**Conclusion:**

For the first time home laundry effluent (HLE) owing to the surfactant like property has been successfully utilised as catalyst for the synthesis of a series of novel 4-thiazolidinone derivatives through one pot, three component condensation-cyclization reaction. The uniqueness of the present protocol lies in the operational simplicity, ability to reduce the demand for organic solvents, reduce the energy and carbon footprint, and meet a wide range of economic needs.

**Electronic supplementary material:**

The online version of this article (doi:10.1186/2193-1801-2-466) contains supplementary material, which is available to authorized users.

## Background

The design, synthesis and development of novel heterocyclic scaffolds of pharmacological importance have fascinated both organic as well as medicinal chemists. Among from the established heterocyclic pharmacophores, 4-thiazolidinone derivatives are deemed to be of considerable importance due to their wide array of biological properties. These derivative posses various pharmacological actions, for instance, antibacterial (Zevzikoviene et al. 2012), anti-HIV (Rawal et al. 2005), antifungal (El Bialy et al. 2011), anticonvulsants (Siddiqui et al. 2010), follicle stimulating hormone (FSH) receptor agonist activity (Wrobel et al. 2006), anti-inflammatory activity (Eleftheriou et al. 2012), anticancer (Havrylyuk et al. 2011), etc. Besides, they are also utilised as chemical antecedents for many compounds, for example polymethine cyanine dyes (El-Aal 2003). More recently, these derivatives were used in the synthesis of pyrazolothiazole derivatives (Turgut et al. 2007) and monofluoro-β lactams (Fuchigami et al. 1992). Consequently, number of protocols have been developed and reported for the synthesis of 4-thiazolidinone including one-step, two-step or via one-pot multicomponent reactions (Kumar et al. 2013). The one-pot multicomponent reactions to synthesize these nuclei offer several advantages and are preferred over traditional synthesis because of their ability to synthesize small drug-like molecules in efficient manner by virtue of minimal workup, high atom economy and being highly modular with several degrees of structural diversity (Orru & de Greef 2003). The reaction involved in synthesis of thiazolidinone proceeds via formation of schiff base intermediate at the initial followed by an attack of a sulphur nucleophile, a mercapto-carboxylic acid, resulting in intermolecular cyclization and eviction of water to yield the desired product (Bolognese et al. 2004). However, removal of the water in the last step is presumably believed as a rate determining step and considered to be critical for obtaining the 4-thiazolidinediones in high yields. As a result, several strategies have been developed for removal of the in situ generated of water for efficient yield of the target molecules. Most commonly followed protocols for water removal involve azeotropic distillation using a Dean Stark trap with either benzene or toluene as solvent medium, molecular sieves and dehydrating agent like DCC (*N,N'*-Dicyclohexylcarbodiimide) (Tierney 1989; Surrey 1947).

However, the conventional protocols for the synthesis of 4-thiazolidinedione have been associated with numerous shortcomings including the use of perilous solvents, expensive catalysts, long work-up procedures, harsh reaction conditions, in-efficient atom economy, and generation of the by-products. More recently, commercially available surfactant *p*-dodecylbenzenesulfonic acid (DBSA) has been reported for synthesis of 2,3-di substituted 4-thiazolidinones derivatives (Prasad et al. 2012). Thus, there has been an urgent need to develop a benign, eco-friendly and inexpensive protocol for the synthesis of 4-thiazolidinone derivatives.

The environmental imbalances have compelled us to adopt the concept of green chemistry in our modern day researches. The green chemistry is a philosophy of chemical research that encourages the design of products and processes that minimize the use and generation of hazardous substances. It aims to protect the environment by inventing new chemical processes that do not pollute, rather than by cleaning up (2012). Water is the most abundant and environmental friendly solvent available in nature and the organic reaction performed in it gained significant attention owing to its various advantages, viz. low cost, safety and environment friendly nature (Dunn 2010). Yet its application in organic synthesis is limited as most organic substrates have poor solubility in water (Shapiro & Vigalok 2008). A good number of strategies have been devised to solve this problem by creating the organic micro-environment in aqueous phase by using surfactants, organic co-solvents or hydrophobic auxiliaries (Lindstrom 2002). On another hand reaction catalysed by surfactants are considered environment benign and are preferred over others (Zhao et al. 2011). Albeit, owing to eco-friendly approach showed by surfactants, its high cost is a major limiting factor for the reactions to be carried out. Therefore, development of novel solvent system which has surfactant like properties at minimal cost is more viable option. Prompted from these ideas, we tried to utilise the surfactant like properties of home laundry effluent (HLE) to catalyse the reactions (Ariel, a laundry detergent (washing powder)). The HLE is categorised as a type of greywater generated from household on washing clothes and has been disposed off in the open environment causing environment pollution. It is entirely different from more heavily contaminated “black water” from toilets. Greywater can be of far higher quality than black water because of its low level of contamination and higher potential for reuse (Allen et al. 2010). Further, use of HLE as reaction media has ability to reduce the demand for organic solvents, reduce the energy and carbon footprint, and meet a wide range of economic needs. This surfactant rich effluent possibly catalyses the reactions through the same way as a surfactant did and provides a greener way of synthesis with economic viability in comparison to the expensive surfactants.

The hybridisation of different pharmacophores in single chemical entity is gaining attraction from the medicinal chemists owing to its synergistic or addition effects, enlarged spectrum of action, less prone to spontaneous mutation and resistance development and possibility of dual drug targeting at more than one site (Morphy & Rankovic 2005). 1,3,5-Triazine, an important heterocyclic scaffold has been found to possess variety of pharmacological activities, such as antibacterial (Gahtori & Ghosh 2012), anti-HIV (Lozano et al. 2011), antifungal (Singh et al. 2012a), antimalarial (Bhat et al. 2013), and anticancer (Bekircan et al. 2005). The hybridisation of this bioactive heterocyclic moiety with another pharmacophores is a matter of investigation of our research programme and till now we have reported numerous hybrid conjugates of 1,3,5-triazine with thiazole (Singh et al. 2011), piperazine (Ghosh et al. 2012), 1,3-thiazine (Singh et al. 2012b) and 1,3,4-thiadiazole (Dubey et al. 2012) as antimicrobial agents.

**Figure 1:**
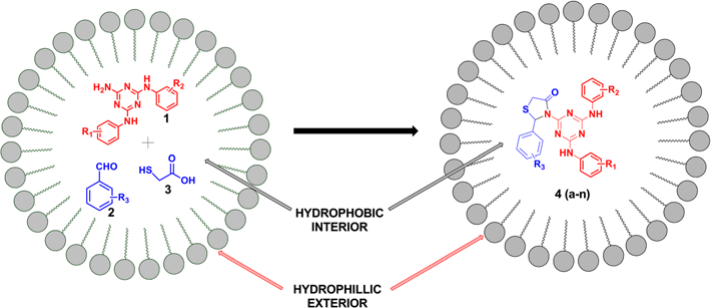
**Synthesis thiazolidine-4-one-1,3,5-triazine catalysed by HLE.**

In continuation of our research endeavour on facile synthesis of novel heterocyclic scaffolds, herein, we report a mild and expeditious aqueous phase one pot synthesis of highly functionalised 4-thiazolidinone incorporated 1,3,5-triazine derivatives catalysed by HLE in moderate to excellent yields, Figure 1.

## Results and discussion

Studies have been carried out to observe the effect of various concentrations of HLE on the reaction as well as its effect on the product yield. As depicted in Table 1, reactions were carried out with and without HLE (blank) at room temperature. The equimolar quantity of amine (*N*^2^,*N*^4^-bis(3-fluorophenyl)-1,3,5-triazine-2,4,6-triamine), aldehyde (2-chlorobenzaldehyde) and thioglycolic acid were taken as model substrates in the presence of 25 mol% of HLE as catalyst at room temperature for 28 h. Surprisingly, a buff white powder of 4f was obtained in 89% yield. It was also revealed that no product was formed in the absence of HLE under the same reaction condition, whereas a 5 mol% of catalyst load resulted in poor yield (10%) even after a prolonged reaction time of 56 h. On further increment of catalyst load to 10 mol%, the product yield was enhanced (25%) in shorter time of 50 h. It is noteworthy that two-fold increase in product yield (4f) accompanied by shorter reaction time was observed on increasing the catalyst load to 15 mol%. A continuous notable shift in product yield (66%) as well as reduction in reaction time (40 h) was observed at the 20 mol% of HLE. As a matter of fact, increase in product yield was accompanied by decreased reaction time under the same reaction condition on each increment of catalytic load till 20 mol% with maximum at 25 mol%, which demonstrated the positive catalytic role of HLE in the synthesis of 4-thiazolidinone. On further increment of catalytic load to 50 mol%, the yield of desired product was decreased to 76%.

**Table 1:** **Catalytic activity evaluation for reaction**^**a**^

Entry	Type of surfactant	Catalyst load	Time (h)	% Yeild^b^
1	HLE	0	No reaction	
2	HLE	5	56	10
3	HLE	10	50	25
4	HLE	15	44	51
5	HLE	20	40	66
6	HLE	25	28	89
7	HLE	50	20	76

*HLE* Home laundry effluent.

^a^Reaction conditions: amine (0.01 mol), aldehyde (0.01 mol), thioglycolic acid (0.01 mol).

^b^Isolated and unoptimised yields.

**Figure 2:**
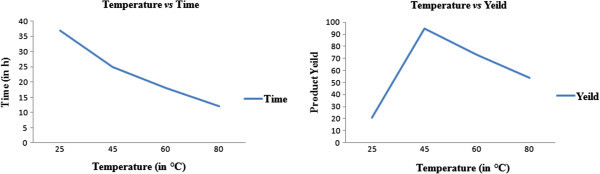
**Effect of temperature on time and yield of the reaction.**

The next phase of the study was aimed to determine the effect of temperature on the reaction. Increasing the temperature, although favoured the cyclization process but did not result in increased product yield. However, an optimum yield of the desired product was obtained at 45°C, Table 2, Figure 2.

**Table 2:** **Effect of temperature on the reaction**^**a**^

Entry	Temp	Time (h)	Yield^b^
1	Room temperature^c^	37	21
2	45	25	95
3	60	18	73
4	80	12	54

^a^Reaction conditions: amine (0.01 mol), aldehyde (0.01 mol), thioglycolic acid (0.01 mol).

^b^Isolated and unoptimised yields.

^c^Average room temperature (25°C).

After optimizing the conditions for cyclization, we intended to determine its scope and limitations for synthesizing hybrid molecules using substituted aldehydes and 1,3,5-triazine derivatives. Under standardised and similar set of reaction conditions, a series of 4-thiazolidinone-1,3,5-triazine conjugates were obtained in fairly good yields ranging from 60-90%. It is shown in Table 3, di-substituted *p*-chloro phenylamine-1,3,5-triazine underwent smooth cyclization on treatment with isomeric chloro and nitro substituted aldehyde derivatives 4(a-d). The compound containing *p*-nitro aldehyde resulted in high yield (4c, 89%), while a drastic decrease in yield was observed with its ortho isomeric counterpart (4d, 79%). Further drop in product yield was observed with chloro substituted aldehyde, the least being in the case of *o*-chloro derivative (4b, 68%). On introduction of fluoro in the place of chloro in 1,3,5-triazine by keeping aldehyde portion constant (4e-4h), a considerably high shift in product yield was observed varying from 81-92%, while the compound containing *o*-NO_2_ substituted aldehyde (4h) showed the lowest yield. However, a notable upward swing in yield was observed on changing the position of NO_2_ to *p*- from *o*- position on aldehyde (92%). It is noteworthy to mention that introduction of non-halogen electron withdrawing group (NO_2_) on di-phenyl amine wings of 1,3,5-triazine along with isomeric chloro substituted aldehyde scaffold resulted in good yield of products (4i-4k). Further a slight increase in reaction time and product yield was observed on insertion of non-halogenated electron withdrawing group (NO_2_) on aldehyde fragment 4k. In the next instance, no substantial change in product yield and reaction condition was observed on altering the substitution pattern of NO_2_ to *p*- position of the phenyl amine linked to 1,3,5-triazine with isomeric chloro substituted aldehydes (4l and 4m). The presence of three NO_2_ groups in the hybrid conjugate (4n) prolonged the reaction time to 35h with marginal change in product yield. Further, presence of bromo group 4(o-r) would not significantly alter the product yield and reaction time. The structures of all newly compounds were ascertained on the basis of IR, ^1^H-NMR, ^13^C-NMR analysis and elemental analysis. The details has been shown in Additional file 1.

**Table 3:** **Synthesis of highly functionalised 4-thiazolidinones catalysed by HLE**

Entry	Amine	Aldehyde	Target Product	Code	Time (in h)	Yield (in %)
1				**4a**	28	71
2				**4b**	25	68
3				**4c**	27	89
4				**4d**	31	79
5				**4e**	28	85
6				**4f**	28	89
7				**4g**	26	92
8				**4h**	28	81
9				**4i**	25	60
10				**4j**	25	72
11				**4k**	29	82
12				**4l**	25	76
13				**4m**	25	69
14				**4n**	35	80
15				**4o**	27	81
16				**4p**	27	76
17				**4q**	27	74
18				**4r**	27	85

It was inferred that halogen containing 1,3,5-traizine and aldehyde derivatives were well suited to this condensation-cyclization reaction with varying reaction time & yield to afford 4-thiazolidine-1,3,5-traizine hybrid conjugates. In general, the substitution on the aldehyde part was found as the main determinant for the reaction time and product yield. It was evident from Table 3 that the reaction proceeded well with aldehyde having the substitution far apart from the reaction centre, *i.e.*, *para* positions (4a, 4c, 4e, 4g, 4i and 4l) and was not favoured with substitution near to reaction centre, *i.e.*, *ortho* positions (4b, 4d, 4f, 4h, 4j, 4k, 4m and 4n). The generation of steric hindrance due to the presence of substituent in the close proximity of reaction centre of aldehyde might be the probable cause of this observation.

**Figure 3:**
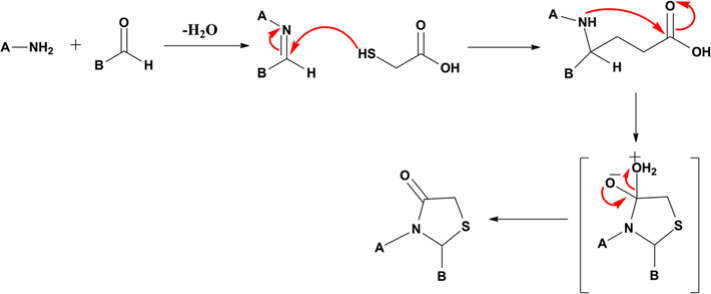
**Mechanism of synthesis thiazolidine-4-one catalysed by HLE, where, A, is amine and B, is aldehyde fragment.**

**Figure 4:**
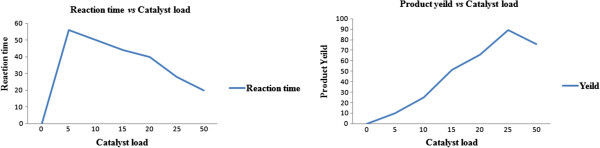
**Effect of catalyst load on reaction time and catalyst load.**

The reaction catalysed by HLE was supposed to follow the classical pseudophase model of the micelles, which may also be called as two domain pseudophase model. It provided the micro-reactor medium for the catalysis. This suggested that the entire system was divided into two reaction domains, *i.e.*, the bulk aqueous region and the entire micellar pseudophase. The reaction was believed to proceed in micellar pseudophase via imine formation (the nitrogen of amine attacks the carbonyl of aldehyde) in the first step followed by attack of sulphur nucleophile on the imine carbon and finally intramolecular cyclization with elimination of water molecule, Figure 3. It was also inferred that the yield of the product was increased on increasing the catalytic load accompanied by shorter reaction time, which may be attributed to increase in number of micelles with increasing concentration of surfactant, Table 1 (Romsted 1977; Jain et al. 2012). However, at an optimum micellar concentration (25 mol%), in which the reactant molecules are almost completely micellized, the increase in micelle concentration (increasing the catalytic load to 50 mol%) does not affect the rate constant of the reaction but rather decreases the rate of the reaction, merely because of the dilution effect on the concentrations of micellized reactants, Figure 4 and serves as the probable cause of decrease in product yield.

### Experimental

#### ***General procedure for synthesis of title hybrid analogues 4 (a-n)***

To a solution of HLE (25 mol%), amine (1, 0.01 mol), aldehyde (2, 0.01 mol) and thioglycolic acid (3, 0.01 mol) were added successively at 45°C and the reaction mixture was stirred for time duration as reported in Table 3. After completion of the reaction, a saturated NaHCO_3_ solution was added followed by the addition of saturated brine solution. The product was extracted with ethyl acetate (3 times). The organic layers were combined, washed with water, dried over anhydrous sodium sulfate and evaporated under reduced pressure to dryness. Purification of crude product was carried out by column chromatography using silica gel (60–120 mesh size) via 10–30% ethyl acetate in heptane as an eluent to furnish the desired product.

## Conclusion

As a concluding remark, for the first time home laundry effluent (HLE) owing to the surfactant like property has been successfully utilised as catalyst for the synthesis of a series of novel 4-thiazolidinone derivatives through one pot, three component condensation-cyclization reaction. The uniqueness of the present protocol lies in the operational simplicity, ability to reduce the demand for organic solvents, reduce the energy and carbon footprint, and meeting a wide range of economic needs. However, the investigations of the characterisation of HLE as catalyst and medicinal properties of these novel compounds are still underway and will be reported in due course of time.

## Electronic supplementary material

Additional file 1: **Experimental details and spectroscopic data of final compounds 4 (a-n).** (DOCX 19 KB)
