# Supplementary material for: Utilisation of Home Laundry Effluent (HLE) as a catalyst for expeditious one-pot aqueous phase synthesis of highly functionalised 4-thiazolidinones
Source: Springerplus. 2013 Sep 16;2:466. doi: 10.1186/2193-1801-2-466 (PMC3786069; doi:10.1186/2193-1801-2-466)
Supplement: Supplementary file 1 — Additional file 1: Experimental details and spectroscopic data of final compounds 4 (a-n). (DOCX 19 KB) [file 40064_2013_522_MOESM1_ESM.docx]

Utilization of Home Laundry Effluent (HLE) as a catalyst for the expeditious one-pot aqueous phase synthesis of highly functionalized 4-thiazolidinones

Udaya Pratap Singh,*^,†,^*^a,c^* Hans Raj Bhat,*^a^* Mukesh Kumar Kumawat,*^b^* Ramendra K. Singh**^c^*

*^a^ Department of Pharmaceutical Sciences, Sam Higginbottom Institute of Agriculture Technology and Sciences, Formerly Allahabad Agricultural Institute, Deemed to be University, Allahabad 211007, India*

*Email:udaysingh98@gmail.com*

*^b^ Anand College of Pharmacy, Agra 282007, India*

*^c^ Nucleic Acids Research Laboratory, Department of Chemistry, University of Allahabad, Allahabad, 211002 India*

*Email: singhramk@rediffmail.com*

**Experimental**

Melting points of the synthesized compounds were determined in an open capillary tube Hicon Melting point apparatus and are uncorrected. Thin layer chromatography (TLC) was performed on silica gel-G coated plates to detect the completion of reaction. The diverse mobile phase was selected in different proportion according to the assumed polarity of the products. The spots was visualised by exposure to the Iodine vapour. Infra-Red (IR) spectra were recorded in KBr on Biored FTs spectrophotometer and the reported wave numbers are given in cm^-1^. ^1^H NMR spectra were recorded in DMSO on Bruker Model D9RX-400MHz spectrometer. Chemical shifts were reported as δ (ppm) relative to TMS as internal standard. Mass spectra were obtained on TOFMS^+^. Elemental analysis of C, H and N was performed on a Vario EL III CHNOS elemental analyzer.

Ariel, a laundry detergent (washing powder) used as surfactant source, product made in India by Procter and Gamble home products Ltd. Mumbai 400099, the common Laundry detergent contains alkylbenzenesulfonates. For this reaction, washed over effluent from the home washing machine of daily use clothes like shirts, pants, scarf’s etc. in mild dirty condition were selected, this liquid was further filtered to remove any solid particles before using as reaction media.

Synthesis of the compounds **1** (**a-d**) was performed in accordance with earlier reported procedures [1].

**General procedure for the synthesis of title hybrid analogues** **4** (**a**-**n**).

To a solution of HLE (25mol%, amines (1, 0.01 mol), aldehydes (2, 0.01 mol) and thioglycolic acid (3, 0.01 mol) were added successively at 45 °C and the reaction mixture was stirred for time as reported in Table 3. After completion of the reaction, a saturated NaHCO_3_ solution was added followed by the addition of saturated brine solution. The product was extracted with ethyl acetate (3 times). The organic layers were combined, washed with water, dried over anhydrous sodium sulfate and evaporated under reduced pressure to dryness. Purification of crude product was carried out by column chromatography using silica gel (60–120 mesh size) via 10–30% ethyl acetate in heptane as an eluent to furnish the desired product.

3-(4,6-Bis((3-chlorophenyl)amino)-1,3,5-triazin-2-yl)-2-(4-chlorophenyl)thiazolidin-4-one. **4a**

Yellowish white powder; M.p.: 244-246 °C; R*f*: 0.56; FT-IR: 3448-3382 (N-H_str_)**,** 3116-2865 (Ar C-H_str_), 1751(C=O_str_), 1693-1626(Ar C=C_str_), 1578-1524(C=N_str_), 1328-1250(Ar C-N_str_), 1016-777(Ar C-H_def_), 869-675(Ar C-Cl_str_); ^1^H-NMR: 2.54 (s, 1H, -NH), 3.22-3.18(d, 1H, *J=* 16 Hz, -NH), 5.31 (s, 1H, -CH), 7.08-7.03 (t, 3H, *J=* 20Hz, Ar-H), 7.32-7.24 (m, 3H, Ar-H), 7.43-7.36 (m, 2H, Ar-H), 7.60-7.57 (d, 2H, *J=* 12 Hz, Ar-H), 7.72 (s, 1H, Ar-H), 7.84 (s, 1H, Ar-H); MS (m/z): 546 [M+H]^+^; Anal. Calcd. For C_24_H_17_Cl_3_N_6_OS: Calculated: C, 53.00; H, 3.15; N, 15.45. Found: C, 53.04; H, 3.10; N, 15.46.

3-(4,6-Bis((3-chlorophenyl)amino)-1,3,5-triazin-2-yl)-2-(2-chlorophenyl)thiazolidin-4-one. **4b**

Yellow powder; M.p. 250-252 °C; R*f*: 0.89; FT-IR: 3292 (N-H_str_), 3116-2860 (Ar C-H_str_), 1751 (C=O_str_), 1684-1622 (Ar C=C_str_), 1573-1517 (C=N_str_), 1330-1248 (Ar C-N_str_), 914-780 (Ar C-H_def_), 780-697 (Ar C-Cl_str_); ^1^H-NMR: 2.14 (s, 1H, -NH), 2.33 (s, 1H, -NH), 7.04-7.02 (d, 2H, *J=*8Hz, Ar-H), 7.11-7.09 (d, 2H, *J=*8Hz, Ar-H), 7.43-7.21 (m, 4H, Ar-H), 7.60 (s, 1H, Ar-H), 7.73 (s, 1H, Ar-H), 7.82 (s, 1H, Ar-H); MS (m/z): 546 [M+H]^+^; Anal. Calcd. for C_24_H_17_Cl_3_N_6_OS: Calculated: C, 53.00; H, 3.15; N, 15.45. Found: C, 53.02; H, 3.14; N, 15.45.

3-(4,6-Bis((3-chlorophenyl)amino)-1,3,5-triazin-2-yl)-2-(4-nitrophenyl)thiazolidin-4-one. **4c**

Brownish Powder; M.p. 239-241°C; R*f*: 0.61; FT-IR: 3273 (N-H_str_), 3111-2927(Ar C-H_str_), 1751(C=O_str_), 1694-1378(Ar C=C_str_), 1579-1544(C=N_str_), 1519(Ar C-NO_2str_), 1353-1276(Ar C-N_str_), 913-673(Ar C-H_def_), 777-617(Ar C-Cl_str_); ^1^H-NMR: 3.46-3.42(t, 1H, *J=*16Hz, -NH), 3.68-3.58(t, 1H, *J=*36Hz, -NH), 7.08-7.02(t, 2H, *J=*24Hz, Ar-H), 7.27-7.26(d, 2H, *J=*4Hz, Ar-H), 7.59-7.57(d, 2H, *J=*8Hz, Ar-H), 7.88-7.67(m, 3H, Ar-H), 8.10-8.08(d, 2H, *J=*8Hz, Ar-H), 8.19-8.17(d, 2H, *J=* 8Hz, Ar-H), 8.26-8.24 (d, 2H, *J=*8Hz, Ar-H); MS (m/z): 554 [M+H]^+^; Anal. Calcd. for C_24_H_17_Cl_2_N_7_O_3_S: Calculated, C, 51.99; H, 3.09; N, 17.68. Found, C, 52.00; H, 3.10; N, 17.65.

3-(4,6-Bis((3-chlorophenyl)amino)-1,3,5-triazin-2-yl)-2-(2-nitrophenyl)thiazolidin-4-one. **4d**

Yellowish Powder; M.p.: 191-193 °C; R*f*: 0.75; FT-IR: 3301(N-H_str_), 3116 (Ar C-H_str_), 1751(C=O_str_), 1622-1570 (Ar C=C_str_), 1519-1481(Ar C-NO_2str_), 1296-1224 (Ar C-N_str_), 990-672 (Ar C-H_def_), 796-607 (Ar C-Cl_str_); ^1^H-NMR: 2.56 (s, 1H, -NH), 3.40(s, 1H, -NH), 7.05-7.03(d, 2H, *J=*8Hz, Ar-H), 7.30-7.26(t, 3H, *J=*16Hz, Ar-H), 7.-92-7.61(m, 4H, Ar-H); MS (m/z): 554 [M+H]^+^; Anal. Calcd. for C_24_H_17_Cl_2_N_7_O_3_S: Calculated, C, 51.99; H, 3.09; N, 17.68. Found, C, 51.96; H, 3.09; N, 17.66.

3-(4,6-Bis((4-fluorophenyl)amino)-1,3,5-triazin-2-yl)-2-(4-chlorophenyl)thiazolidin-4-one. **4e**

Brownish black powder; M.p.: 302-303°C; R*f*: 0.60; FT-IR: 3224(N-H_str_), 3115-2933 (Ar C-H_str_), 1753 (C=O_str_), 1686 (Ar C=C_str_), 1376 (Ar C-N_str_), 1014-1091 (C-F_str_), 759-833 (Ar C-H_def_); ^1^H-NMR: 2.57 (s, 1H, -NH), 3.19-3.15 (d, 1H, *J=*16Hz, -NH), 7.13-7.02 (m, 2H, Ar-H), 7.22 (s, 1H, Ar-H), 7.34-7.33 (d, 2H, *J=*8Hz, Ar-H), 7.43-7.41 (d, 2H, *J=*8Hz, Ar-H), 7.61 (s, H, Ar-H), 7.79 (s, 1H, Ar-H), 7.98 (s, 1H, Ar-H); MS (m/z): 511 [M+H]^+^; Anal. Calcd. for C_24_H_17_ClF_2_N_6_OS: Calculated, C, 56.42; H, 3.35; N, 16.45. Found, C, 56.44; H, 3.38; N, 16.47

3-(4,6-Bis((4-fluorophenyl)amino)-1,3,5-triazin-2-yl)-2-(2-chlorophenyl)thiazolidin-4-one. **4f**

Brownish black powder; M.p.: 289-291 °C; R*f*: 0.80; FT-IR: 3220 (N-H_str_), 3119-2875 (Ar C-H_str_), 1756 (C=O_str_), 1686-1595 (Ar C=C_str_), 1322-1296 (Ar C-N_str_), 1125-1013 (C-F_str_), 1158 (C-N_str_), 874-727 (Ar, C-H_def_), 752-727 (Ar C-Cl_str_); ^1^H-NMR: 3.21(s, 1H, -N-H), 3.25 (s, 1H, -N-H), 7.16-6.99 (m, 4H, Ar-H), 7.39-7.21 (m, 2H, Ar-H), 7.47-7.44 (t, 2H, *J=* 12Hz , Ar-H), 7.52-7.50 (d, 2H, *J=*8Hz, Ar-H), 7.59 (s, 1H, Ar-H), 7.88 (s, 1H, Ar-H); MS (m/z): 511 [M+H]^+^; Anal. Calcd. for C_24_H_17_ClF_2_N_6_OS: Calculated, C, 56.42; H, 3.35; N, 16.45. Found, C, 56.40; H, 3.35; N, 16.46.

3-(4,6-Bis((4-fluorophenyl)amino)-1,3,5-triazin-2-yl)-2-(4-nitrophenyl)thiazolidin-4-one. **4g**

Brown powder; M.p.: >360 °C; R*f*: 0.70; FT-IR: 3286-3281(N-H_str_), 3118-2872 (Ar C-H_str_), 1755 (C=O_str_), 1687-1503 (Ar C=C_str_), 1417 (Ar C-NO2_str_), 1342 (Ar C-N_str_), 1011 (C-F_str_), 895-673 (Ar C-H_def_); ^1^H-NMR: 3.24-3.20(d, 1H, *J=*16Hz, -N-H), 3.44-3.40(d,1H, *J=*16Hz, -NH), 7.05-7.01(t, 2H, *J=*16Hz, Ar-H), 8.01(s, 1H, Ar-H), 8.21-8.19(d, 2H, *J=*12Hz, Ar-H); MS (m/z): 522 [M+H]^+^; Anal. Calcd. for C_24_H_17_F_2_N_7_O_3_S: Calculated, C, 55.27; H, 3.29; N, 18.80; Found, C, 55.30; H, 3.28; N, 18.85.

3-(4,6-Bis((4-fluorophenyl)amino)-1,3,5-triazin-2-yl)-2-(2-nitrophenyl)thiazolidin-4-one. **4h**

Whitish powder; M.p.: 293-295 °C; R*f*: 0.85; FT-IR: 3220(N-H_str_), 3118-2872 (Ar C-H_str_), 1756 (C=O_str_), 1687-1595 (Ar C=C_str_), 1491(Ar C-NO_2str_),1320-1293 (Ar C-N_str_), 1012 (C-F_str_), 833-725 (Ar C-H_def_); ^1^H-NMR: 3.24 (s, 1H, -N-H), 3.30-3.26 (t, 1H, *J=*16Hz, -N-H), 7.14-7.02 (m, 4H, Ar-H), 7.22-7.20 (d, 2H, *J=*8Hz, Ar-H), 7.33 (s, 1H, Ar-H), 7.75-7.71 (t, 2H, *J=*16Hz, Ar-H); MS (m/z): 522 [M+H]^+^; Anal. Calcd. for C_24_H_17_F_2_N_7_O_3_S: Calculated, C, 55.27; H, 3.29; N, 18.80; Found, C, 55.28; H, 3.30; N, 18.82.

3-(4,6-Bis((3-nitrophenyl)amino)-1,3,5-triazin-2-yl)-2-(4-chlorophenyl)thiazolidin-4-one. **4i**

Yellowish white powder; M.p.: 131-133 °C; R*f*: 0.84; FT-IR: 3354 (N-H_str_), 3096 (Ar C-H_str_), 1697 (C=O_str_), 1638-1566 (Ar C=C_str_), 1526 (Ar C-NO_2_str), 1347-1301 (Ar C-Nstr), 883-734 (Ar C-H_str_), 618-796 (Ar C-C_str_); ^1^H-NMR: 2.10 (s, 1H, -N-H), 2.31 (s, 1H, -N-H), 7.41-7.36 (m, 3H, Ar-H), 7.59-7.49 (m, 3H, Ar-H), 7.96-7.83 (m, 2H, Ar-H), 8.18 (s, 1H, Ar-H), 8.64-8.61 (d, 2H, *J=*12Hz, Ar-H); MS (m/z): 565 [M+H]^+^; Anal. Calcd. for C_24_H_17_ClN_8_O_5_S: Calculated, C, 51.02; H, 3.03; N, 19.83, Found, C, 51.04; H, 3.06; N, 19.80.

3-(4,6-Bis((3-nitrophenyl)amino)-1,3,5-triazin-2-yl)-2-(2-chlorophenyl)thiazolidin-4-one. **4j**

Brown powder; M.p.: 260-262 °C; R*f*: 0.70; FT-IR: 3328 (N-H_str_), 3123-2974 (Ar C-H_str_), 1759 (C=O_str_), 1670-1561 (Ar C=C_str_), 1526 (Ar C-NO_2str_), 1348-1301 (Ar C-N_str_), 889-736 (Ar C-H_def_), 677 (Ar C-Cl_str_); ^1^H-NMR: 3.22 (s, 1H, -NH), 3.26 (s, 1H, -N-H), 7.15-7.13 (d, 2H, *J=*8Hz, Ar-H), 7.42-7.27 (m, 3H, Ar-H), 7.54-7.48 (m, 3H, Ar-H), 8.09 (s, 1H, Ar-H); MS (m/z): 565 [M+H]^+^; Anal. Calcd. for C_24_H_17_ClN_8_O_5_S: Calculated, C, 51.02; H, 3.03; N, 19.83, Found, C, 51.01; H, 3.00; N, 19.86.

3-(4,6-Bis((3-nitrophenyl)amino)-1,3,5-triazin-2-yl)-2-(2-nitrophenyl)thiazolidin-4-one. **4k**

Pinkish powder; M.p.: 222-244 °C; R*f*: 0.64; FT-IR: 3386 (N-H_str_), 3080 (Ar C-H_str_), 1701 (C=O_str_), 1614-1576 (Ar C=C_str_), 1513 (Ar C-NO_2str_), 1349 (Ar C-N_str_), 885-735 (Ar C-H_def_); ^1^H-NMR: 2.14 (s, 1H, -N-H), 2.33 (s, 1H, -N-H), 7.53-7.42 (m, 4H, Ar-H), 7.78-7.76 (m, 3H, Ar-H), 7.93-7.83 (m, 3H, Ar-H), 8.69 (s, 1H, Ar-H); MS (m/z): 576 [M+H]^+^; Anal. Calcd. for C_24_H_17_ClN_9_O_7_S: Calculated, C, 50.09; H, 2.98; N, 21.90, Found, C, 50.05; H, 2.97; N, 21.94.

2-(4-Chlorophenyl)-3-(4-((3-nitrophenyl)amino)-6-((4-nitrophenyl)amino)-1,3,5-triazin-2-yl)thiazolidin-4-one. **4l**

Black powder: M.p.: 145-147 °C; R*f*: 0.84; FT-IR: 3367 (N-H_str_), 2915 (Ar C-H_str_), 1629-1596 (Ar C=Cstr), 1692 (C=O_str_), 1479 (Ar C-NO_2str_), 1305 (C-N_str_), 891-696 (Ar C-H_def_), 751-631(Ar C-Cl_str_); ^1^H-NMR: 2.33(s, 1H, -NH), 2.57(s, 1H, -NH), 6.63-6.61(m, 3H, Ar-H), 7.59-7.54(m, 2H, Ar-H),7.99-7.87(m, 2H, Ar-H); MS (m/z): 565 [M+H]^+^; Anal. Calcd. for C_24_H_17_ClN_8_O_5_S: Calculated, C, 51.02; H, 3.03; N, 19.83, Found, C, 51.04; H, 3.06; N, 19.80.

2-(2-Chlorophenyl)-3-(4-((3-nitrophenyl)amino)-6-((4-nitrophenyl)amino)-1,3,5-triazin-2-yl)thiazolidin-4-one. **4m**

Black powder: M.p.: 150-152 °C; R*f*: 0.80; FT-IR: 3353 (N-H_str_), 3193-2864 (Ar C-H_str_), 1700 (C=O_str_), 1599 (Ar C=C_str_), 1526 (Ar C-NO_2str_), 1345-1244 (Ar C-N_str_), 798-735 (Ar C-H_def_), 668 (Ar C-Cl_str_); ^1^H-NMR: 3.47 (s, 1H, -NH), 3.80 (s, 1H, -NH), 5.76 (s, 1H, Ar-H), 6.80-6.60 (m, 4H, Ar-H), 7.18-7.15 (m, 3H, Ar-H), 7.37-7.34 (m, 2H, Ar-H); MS (m/z): 565 [M+H]^+^; Anal. Calcd. for C_24_H_17_ClN_8_O_5_S: Calculated, C, 51.02; H, 3.03; N, 19.83, Found, C, 51.03; H, 3.03; N, 19.83.

2-(2-Nitrophenyl)-3-(4-((3-nitrophenyl)amino)-6-((4-nitrophenyl)amino)-1,3,5-triazin-2-yl)thiazolidin-4-one. **4n**

Dark brown powder; M.p.: 200-202 °C; R*f*: 0.77; FT-IR: 3355 (N-H_str_), 3082-2921 (Ar C-H_str_), 1699 (C=O_str_), 1629-1598 (Ar C=C_str_), 1526-1432 (Ar C-NO_2str_), 1341-1304 (Ar C-N_str_),733-798 (Ar C-H_def_); ^1^H-NMR: 2.33 (s, 1H, -N-H), 2.58 (s, 1H, -N-H), 6.09-6.03 (d, 2H, *J=*24Hz, Ar-H), 6.24 (s, 1H, Ar-H), 7.22-7.14 (m, 3H, Ar-H), 7.50-7.42 (m, 4H, Ar-H), 8.70 (s, 1H, Ar-H); MS (m/z): 576 [M+H]^+^; Anal. Calcd. for C_24_H_17_N_9_O_7_S: Calculated, C, 50.09; H, 2.98; N, 21.90, Found, C, 50.06; H, 3.01; N, 21.94.

3-(4,6-Bis((3-bromophenyl)amino)-1,3,5-triazin-2-yl)-2-(4-chlorophenyl)thiazolidin-4-one. **4o**

Brown powder; M.p.: 252-253 °C; R*f*: 0.58; FT-IR: 3449-3385 (N-H_str_)**,** 3118-2862 (Ar C-H_str_), 1756 (C=O_str_), 1693-1628 (Ar C=C_str_), 1576-1531(C=N_str_), 1326-1252 (Ar C-N_str_), 1018-778 (Ar C-H_def_), 845-625(Ar C-Br_str_); ^1^H-NMR: 2.58 (s, 1H, -NH), 3.28-3.13(d, 1H, *J=* 12 Hz, -NH), 5.45 (s, 1H, -CH), 7.18-7.05 (t, 3H, *J=* 16 Hz, Ar-H), 7.37-7.22 (m, 3H, Ar-H), 7.48-7.39 (m, 2H, Ar-H), 7.61-7.56 (d, 2H, *J=* 12 Hz, Ar-H), 7.76 (s, 1H, Ar-H), 7.87 (s, 1H, Ar-H); MS (m/z): 633 [M+H]^+^; Anal. Calcd. For C_24_H_17_Br_2_ClN_6_OS: Calculated: C, 45.56; H, 2.71; N, 13.28. Found: C, 45.56; H, 2.70; N, 13.28.

3-(4,6-Bis((3-bromophenyl)amino)-1,3,5-triazin-2-yl)-2-(2-chlorophenyl)thiazolidin-4-one. **4p**

Dark brown powder; M.p. 258-260 °C; R*f*: 0.76; FT-IR: 3294 (N-H_str_), 3119-2865 (Ar C-H_str_), 1756 (C=O_str_), 1694-1628 (Ar C=C_str_), 1575-1528 (C=N_str_), 1328-1252 (Ar C-N_str_), 918-786 (Ar C-H_def_), 785-692 (Ar C-Cl_str_), 845-625 (Ar C-Br _str_); ^1^H-NMR: 2.15 (s, 1H, -NH), 2.35 (s, 1H, -NH), 7.08-7.02 (d, 2H, *J=*6 Hz, Ar-H), 7.18-7.06 (d, 2H, *J=*6 Hz, Ar-H), 7.48-7.18 (m, 4H, Ar-H), 7.68 (s, 1H, Ar-H), 7.78 (s, 1H, Ar-H), 7.84 (s, 1H, Ar-H); MS (m/z): 633 [M+H]^+^; Anal. Calcd. for C_24_H_17_Br_2_ClN_6_OS: Calculated: C, 45.56; H, 2.71; N, 13.28. Found: C, 45.55; H, 2.71; N, 13.28.

3-(4,6-bis((3-bromophenyl)amino)-1,3,5-triazin-2-yl)-2-(4-nitrophenyl)thiazolidin-4-one. **4q**

Yellow brownish powder; M.p. 232-234°C; R*f*: 0.68; FT-IR: 3275 (N-H_str_), 3113-2928 (Ar C-H_str_), 1751(C=O_str_), 1698-1382 (Ar C=C_str_), 1576-1542 (C=N_str_), 1518(Ar C-NO_2str_), 1358-1278(Ar C-N_str_), 916-675(Ar C-H_def_), 845-625 (Ar C-Br _str_); ^1^H-NMR: 3.48-3.44(t, 1H, *J=*14Hz, -NH), 3.69-3.61(t, 1H, *J=*18 Hz, -NH), 7.12-7.04 (t, 2H, *J=*12 Hz, Ar-H), 7.27-7.26(d, 2H, *J=*4Hz, Ar-H), 7.62-7.57(d, 2H, *J=*8Hz, Ar-H), 7.87-7.64 (m, 3H, Ar-H), 8.12-8.10(d, 2H, *J=*6 Hz, Ar-H), 8.21-8.16(d, 2H, *J=* 8Hz, Ar-H), 8.28-8.25 (d, 2H, *J=*6Hz, Ar-H); MS (m/z): 644 [M+H]^+^; Anal. Calcd. for C_24_H_17_Br_2_N_7_O_3_S: Calculated, C, 44.81; H, 2.66; N, 15.24. Found, C, 44.82; H, 2.66; N, 15.24.

3-(4,6-bis((3-bromophenyl)amino)-1,3,5-triazin-2-yl)-2-(2-nitrophenyl)thiazolidin-4-one. **4r**

Light Yellowish brown powder; M.p.: 215-217 °C; R*f*: 0.68; FT-IR: 3305 (N-H_str_), 3112 (Ar C-H_str_), 1753 (C=O_str_), 1628-1565 (Ar C=C_str_), 1523-1487 (Ar C-NO_2str_), 1292-1229 (Ar C-N_str_), 991-675 (Ar C-H_def_), 845-625 (Ar C-Br _str_); ^1^H-NMR: 2.58 (s, 1H, -NH), 3.43 (s, 1H, -NH), 7.06-7.04 (d, 2H, *J=*8Hz, Ar-H), 7.35-7.28 (t, 3H, *J=*12 Hz, Ar-H), 7.-98-7.65 (m, 4H, Ar-H); MS (m/z): 644 [M+H]^+^; Anal. Calcd. for C_24_H_17_Br_2_N_7_O_3_S: Calculated, C, 44.81; H, 2.66; N, 15.24. Found, C, 44.81; H, 2.67; N, 15.24.
